# A multiple cavity malignancy involving the renal capsule, pleura and meninges: A case report and review of the literature

**DOI:** 10.3892/ol.2013.1451

**Published:** 2013-07-09

**Authors:** LI-JING ZHU, BAO-RUI LIU, XIAO-PING QIAN, WEI-WEI KONG, WEN-JING HU, JUAN DU, HAI-QING ZHU

**Affiliations:** 1 The Comprehensive Cancer Center, Drum Tower Hospital, Medical School of Nanjing University, Clinical Cancer Institute of Nanjing University, Nanjing, Jiangsu 2l0008, P.R. China; 2 Department of Pathology, Nanjing Brain Hospital, Nanjing, Jiangsu 2l0029, P.R. China

**Keywords:** neoplasms, unknown primary site, pleural effusion, renal, adenocarcinoma

## Abstract

Malignant renal subcapsular effusions commonly arise from primary or metastatic renal neoplasms. The current case report presents a rare case of malignancy with a massive renal subcapsular effusion accompanied by a malignant pleural effusion of an unknown primary site, which underwent progression to carcinomatous meningitis during chemotherapy. The type of adenocarcinoma present was determined by effusion cytology. Intravenous chemotherapy (docetaxel plus oxaliplatin and gemcitabine plus cisplatin) were administered; however, the disease still progressed. Time to progression was 9 months during treatment of gefitinib. Comprehensive therapies, including intracavity chemotherapy, immunotherapy and gefitinib, were shown to be effective and prolonged the patient’s survival time.

## Introduction

Cancer of unknown primary site (CUP) is a category of malignancy with undetectable primary site upon diagnosis. It is characterized by diverse clinical manifestations and poor prognosis (1). The histological types include adenocarcinoma, squamous cell carcinoma, neuroendocrine tumors and carcinoma not otherwise specified. Adenocarcinoma of unknown primary site accounts for 60–70% of all cases of CUP and is commonly identified in the liver, lung, bone and lymph nodes (1,2). Metastatic adenocarcinoma of unknown primary site, first presenting with malignant renal subcapsular effusions, is extremely rare and has not been analyzed in previous studies. The current case report presents a patient with adenocarcinoma of unknown primary site that initially presented with renal subcapsular and pleural effusion followed by carcinomatous meningitis. The treatment of this specific type of carcinoma is subsequently discussed. Written informed consent was obtained from the patient.

## Case report

### Patient presentation and diagnosis

**Figure 1:**
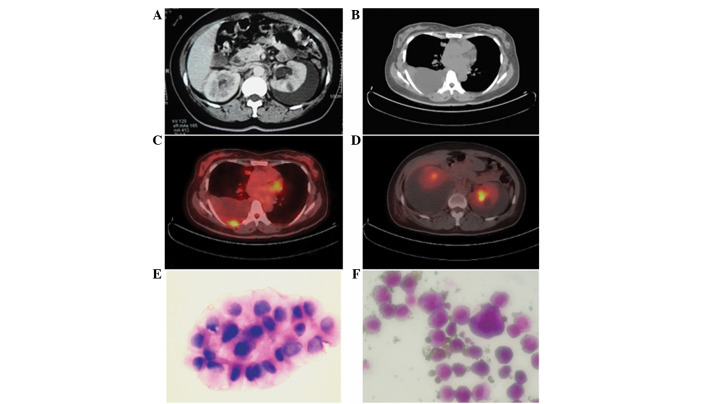
Radiological and cytopathological manifestations of metastatic adenocarcinoma of an unknown primary site. (A) Abdominal contrast-enhanced computed tomography (CT) scan (March 7, 2006) showing a cyst, renal subcapsular effusion and mild hydrocele in the left kidney. (B) Mediastinal window of the CT scan one month later (April 10, 2006) showing a solid nodule in the upper right lung. Positron emission tomography (PET)/CT (April 10, 2006) showing (C) bilateral subcapsular and (D) right pleural effusions. The site of increased ^18^F-fluorodeoxyglucose (FDG) uptake indicates the puncture point between the ribs. Cytology of the (E) subcapsular effusion, as determined by hematoxylin and eosin staining, and cytology of the (F) cerebrospinal fluid, as determined by Wright’s stain, showing large numbers of adenocarcinomatous cells.

A 45-year-old female was admitted to The Comprehensive Cancer Center, Drum Tower Hospital (Nanjing, China) on April 9, 2006 with flank pain, cough and dyspnea. One month prior to admission, the patient had suffered from left flank pain. Abdominal ultrasonography at the patient’s local hospital revealed a subcapsular effusion, a small cyst in the left kidney and a calculus of 0.8 cm in diameter in the upper right ureter. The patient was treated for lithiasis for ~1 month with no amelioration and subsequently developed a cough and dyspnea. A computed tomography (CT) scan revealed right pleural and subcapsular effusions in the left kidney (Fig. 1A). A thorough examination of the individual was performed, including an intravenous urogram, mammography, gynecological ultrasonography, whole gastrointestinal barium contrast imaging and colonoscopy, however, no abnormalities were identified.

Upon admission, a physical examination revealed high blood pressure (160/100 mmHg), lowered respiratory sounds in the right lung and a mass in the left loin. Blood tests showed an elevated level of serum creatinine (196 μmol/l) and serum tumor markers, cancer antigen (CA)125 (168 U/ml; reference range, <35 U/ml), carcinoembryonic antigen (CEA; 15.3 ng/ml; reference range, <5.0 ng/ml) and CA153 (68 U/ml; reference range, <25 U/ml) and a urine test was negative. The cytopathological examination of the pleural effusion was positive for poorly-differentiated adenocarcinoma cells. To identify the primary tumor site, the patient received a systemic positron emission tomography (PET)/CT scan, which identified a small nodule measuring 7×12×9 mm in the upper lobule of the right lung and right pleural (Fig. 1B and C) and subcapsular effusions of the kidneys (Fig. 1D), with normal ^18^F-fluorodeoxyglucose (FDG) uptake. Due to its size and site, a fine-needle biopsy of the lung nodule was not performed and therefore, the patient was diagnosed with a carcinoma of unknown primary site.

### Treatment and clinical course

**Figure 2:**
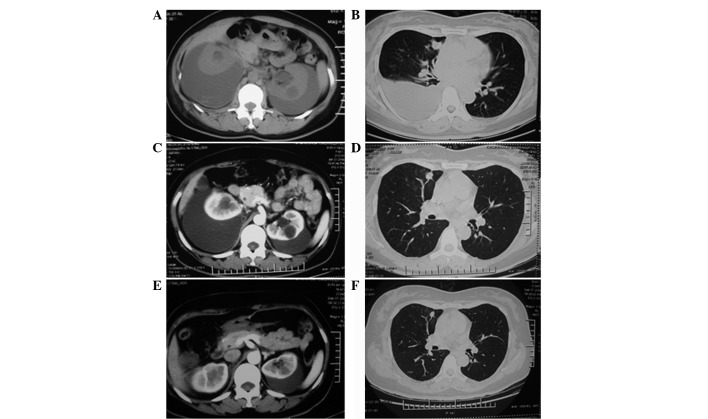
(A and B) CT scans prior to treatment (April 10, 2006) showing bilateral renal subcapsular effusion, a cyst in the kidney, a right pleural effusion and a nodule in the upper lobule of the right lung. (C and D) Following three cycles of intravenous chemotherapy and intra-pleural immunotherapy (June 23, 2006) the pleural effusion subsided but the nodule in the right lung and the subcapsular effusions did not change. (E and F) Following an additional three cycles of intravenous and subcapsular chemotherapy and immunotherapy (September, 7 2006), the subcapsular effusion significantly decreased and no apparent changes to the lung nodule were observed.

Chemotherapy was initiated and agents with high renal toxicity were excluded to avoid the deterioration of the patient’s kidney function. Intravenous docetaxel (40 mg/m^2^ on days 1 and 8, every 3 weeks) plus oxaliplatin (75 mg/m^2^ on days 1 and 8, every 3 weeks) and intrapleural interleukin-2 (1×10^9^ U on days 3 and 10, every 3 weeks) was administered. The pleural effusion subsided after being drained three times and intrapleural interleukin-2 administration. Following the initial course of chemotherapy, the cough and dyspnea ameliorated and serum levels of creatinine and tumor markers decreased. Following three cycles of chemotherapy, the CT scan showed no change in the pulmonary nodule (Fig. 2B and D) or subcapsular effusion (Fig. 2A and C). Next, renal subcapsular drainage was performed and ~330 and 960 ml stale hematoid fluid was aspirated from the left and right sides, respectively. The levels of CA125, CA199 and CA153 tumor markers were significantly elevated in the drained fluid and adenocarcinoma cells were evident upon analysis of the drainage cytology (Fig. 1E). Following sequential treatments of interleukin-2 and bleomycin administered intrasubcapsularly, the amount of drained fluid decreased (Fig. 2C and E) and turned yellow and clear. The abdominal symptoms were ameliorated and the patient’s blood pressure and serum creatinine level returned to normal; however, no clear changes to the lung nodule were observed (Fig. 2F). Subsequently, the patient received an additional three cycles of systemic chemotherapy with gemcitabine (1,000 mg/m^2^ on days 1 and 8, every 3 weeks) plus cisplatin (25 mg/m^2^ daily for 3 days ).

The patient’s condition was stable prior to an intense headache that developed three weeks after the final administration of gemcitabine plus cisplatin. The individual exhibited no vomiting or blurred vision, the blood pressure remained normal and the brain and spinal MRI scans were negative. A lumber puncture was performed and the cerebrospinal fluid was positive for a large number of adenocarcinoma cells (Fig. 1F). Immunostaining was positive for epithelial membrane antigen and pan-cytokeratin, but negative for vimentin, glial fibrillary acidic protein, cluster of differentiation (CD)3, CD20, CD30 and CD68. Meningeal metastasis and carcinomatous meningitis were diagnosed and the patient received 10 mg intrathecal methotrexate weekly, but refused cranial and spinal cord radiation therapy. Following a total of 40 mg intrathecal methotrexate chemotherapy, the patient’s headache was ameliorated and the cerebrospinal fluid was negative for tumor cells. The individual was administered with 250 mg gefitinib daily for the following nine months, during which the disease status was stable. However, following this, the patient developed paroxysmal syncope and epileptic seizures. No abnormalities were identified in the cranial MRI. Following an additional four months of treatment, the patient succumbed to carcinoma of unknown primary site on March 20, 2008, ~23.5 months after the initial diagnosis. An autopsy was not performed according to the wishes of the patient’s family.

## Discussion

CUP comprises 2–5% of all diagnosed tumors (1,2). It represents a heterogeneous group of metastatic tumors that share unique clinical features, including early dissemination, a clinical absence of a primary tumor, unpredictable metastatic patterns and aggressiveness, and patients tend to have an unfavorable prognosis. Adenocarcinoma of unknown primary site commonly presents in the liver, lungs, lymph nodes and bones and is rarely identified with malignant pleural or peritoneal effusions (3). A malignant renal subcapsular effusion of unknown primary site is extremely rare and has not been analyzed in previous studies. The current case report presents an unusual manifestation of CUP with massive malignant renal subcapsular and pleural effusions and subsequent carcinomatous meningitis. No solid metastasis was identified during a thorough examination.

Renal subcapsular effusions may be hydroceles or hematoceles, and various mechanisms, including venous, lymphatic and urine regurgitation, may contribute to a subcapsular hydrocele of the kidney. However, a hematocele is likely to be caused by trauma, a renal tumor, vascular disease, infection, nephritis, blood dyscrasias, calculus or hydronephrosis (4). With an incidence rate of 57.7%, spontaneous subcapsular or perirenal hematomas are commonly associated with primary renal neoplasms, of which 33.4% are malignant with renal cell carcinoma predominance and 24.3% are benign with angiomyolipoma predominance (5). In addition, tumors occasionally arise from metastasis of extrarenal primary tumors to the kidney (6). In the present case report, the subcapsular effusion was hematoid and positive for adenocarcinoma cells. The renal parenchyma was negative for any discernible lesions with the exception of a cyst in the left kidney, which did not resemble a common entity of a metastasis of adenocarcinoma cells and did not change throughout the duration of the treatment process. In addition, the urine test was negative for tumor cells or hematuria. As the effusion was bilateral, subcapsular metastasis from the extrarenal site was hypothesized.

No consensus has been made with regard to the standard therapy for CUP and particularly for multiple malignant cavity effusions. Empiric chemotherapy with 5-fluorouracil, doxorubicin or cisplatin-based regimens have previously produced relatively low response rates and few complete responses (7). Broad spectrum antineoplastic agents, including taxanes, topoisomerase I inhibitors, gemcitabine and vinorelbine, have been investigated in epithelial CUP, and platinum and taxane combination therapy is now widely used in clinical practice. However, a previous meta-analysis showed that no types of chemotherapy have been confirmed to prolong survival in patients with CUP (8). Paclitaxel/docetaxel-containing combination regimens have been used in specific phase II trials and the preliminary results have shown response rates between 23 and 38.7% (9–11). Briasoulis *et al* reported encouraging results from phase II data on carboplatin and paclitaxel combination therapy for patients with CUP (12). The overall response rate by an intention-to-treat analysis was 38.7% and the median overall survival time was 13 months with a median follow-up of 28 months. A recent randomized study compared empiric therapy with paclitaxel/carboplatin/etoposide against gemcitabine/irinotecan, each followed by single-agent gefitinib, and subsequently identified a comparable efficacy. However, gemcitabine/irinotecan therapy revealed a more favorable toxicity profile (13). In the current case report, the docitaxel/oxiplatin and gemcitabine/cisplatin regimens were administered to the patient successively, but the efficacy was limited since the tumor rapidly metastasized to the meninges and caused carcinomatous meningitis.

As a maintenance therapy, gefitinib was effective and the patient’s condition was stabilized for nine months despite metastasis of the tumor to the meninges and subsequent carcinomatous meningitis, for which the median survival time is 2–3 months. Patients untreated or unresponsive to treatment exhibit a median survival of 4–6 weeks (14,15). Overexpression of epidermal growth factor receptor (EGFR) has been observed frequently in a large subset of CUP and gefitinib is effective in a broad spectrum of tumor types (16–19). Although no previous randomized studies have analyzed the effect of gefitinib in CUP, a previous prospective study reported that the combination of vascular endothelial growth factor receptor inhibitor (bevacizumab) and EGFR inhibitor (erlotinib) in CUP has a better median survival than previously reported with second-line chemotherapy and is similar to the results of a number of first-line therapies (20). Gefitinib maintenance therapy was also included in a recent prospective randomized trial (13). The role of gefitinib in adenocarcinoma with CUP is promising and must be analyzed in future studies.

In conclusion, the current case report presents the case of a patient with a malignant effusion in multiple cavities and demonstrates the intracavity administration of chemotherapeutic agents. Interleukin-2 appeared to be effective for controlling the effusion, however, systemic chemotherapy with docetaxel plus oxaliplatin, followed with gemcitabine plus cisplatin, appeared non-effective and the patient exhibited disease progression with involvement of the central nervous system, indicating the refractory entity and poor prognosis of this type of carcinoma. The overall survival time of the patient was ~24 months, which was considerably higher than the normal median survival of individuals with CUP. Gefitinib was identified to be promising for maintenance therapy and was shown to prolong the patient’s survival.
